# 808. Appropriate Use of Cephalotin Before and After Implementation of a Cardiac Surgery Antibiotic Prophylaxis Protocol in Guatemala

**DOI:** 10.1093/ofid/ofab466.1004

**Published:** 2021-12-04

**Authors:** Herberth G Maldonado, Brooke M Ramay, Lourdes A Sandoval

**Affiliations:** 1 Unidad de Cirugía Cardiovascular de Guatemala, Guatemala, Quetzaltenango, Guatemala; 2 Universidad del Valle de Guatemala, Center for Health Studies, Paul G. Allen School for Global Health, Washington State University, Pullman, USA, Guatemala City, Sacatepequez, Guatemala; 3 Abbott, Guatemala City, Baja Verapaz, Guatemala

## Abstract

**Background:**

The appropriate use of Surgical Antibiotic Prophylaxis (SAP) contributes to reducing the prevalence of Surgical Site Infections (SSI). Inappropriate use increases the risk of SSIs, hospitalization costs and potentially contributes to the emergence of antimicrobial resistance. We aimed to compare the appropriate use before and after implementing a SAP protocol in our institution

**Methods:**

We conducted a retrospective chart review in patients older than 18 undergoing elective cardiac surgery with cardiopulmonary bypass using cephalotin as SSI prophylaxis. We excluded patients who received other antimicrobials for prophylaxis, those undergoing non-elective surgery, and patients with delayed sternal closure. We identified SSIs according to the Centers for Disease Prevention and Control criteria. We evaluated if appropriate dosing (2g-3g) and timing ( >60 min.) occurred before the surgical incision, if redosing was administered, and if prophylaxis was administered > 48 hours. We evaluated before and after implementation of the protocol (August 2016-July 2017; October 2017-2018)

**Results:**

The study included 262 and 285 patients before and after protocol implementation, respectively. Patient characteristics were similar between comparator groups (Table 1). We found that 1.1% of patients vs. 63% of patients had appropriate dosing before the surgical incision, before and after protocol implementation, respectively (p < 0.05). There was no difference in appropriate redosing when the duration of surgery was greater than 4 hours and no difference in inappropriate prophylaxis administration > 48 hours after protocol implementation. A total of 8 SSIs were identified in each group, with no statistical difference in the incidence, length of stay, or clinical outcome between comparator groups

Table 1. Patient Characteristics and Appropriate use of Cephalotin Before and After Implementation of a Cardiac Surgery Antibiotic Prophylaxis Protocol in Guatemala

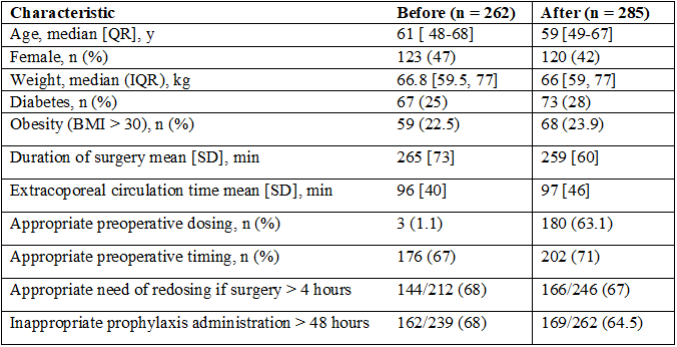

**Conclusion:**

Based on our findings, implementing a local guideline-protocol for SAP resulted in significant improvement of pre-surgical antimicrobial dosing. We observed continual unnecessary administration of antibiotic prophylaxis in the postoperative period that needs more proactive interventional pharmacy-guided strategies such as automatic stops or audits width feedback.

**Disclosures:**

**Lourdes A. Sandoval, Master of Science in Pharmacovigilance and Pharmacoepidemiology**, **Abbott** (Employee)

